# Quality of life and sexual health after perineal reconstruction in Fournier gangrene using pedicled anterolateral thigh flaps

**DOI:** 10.3389/fsurg.2022.994936

**Published:** 2022-09-13

**Authors:** Severin Alexander Rossi, Camille de Schoulepnikoff, David Guillier, Wassim Raffoul, Pietro Giovanni di Summa

**Affiliations:** ^1^ Unit of Plastic and Hand Surgery, CHUV University hospital of Lausanne – UNIL, University of Lausanne, Lausanne, Switzerland; ^2^ Unit of Plastic and Hand Surgery, Hôpital le Bocage CHRU Dijon, Dijon, France

**Keywords:** fournier gangrene, reconstruction, pedicled anterolateral thigh flap, perineal defect, sexual wellbeing, quality of life

## Abstract

**Objectives:**

To assess long-term sexual outcome and quality of life after perineal reconstruction by pedicled anterolateral thigh (ALT) flaps after Fournier's gangrene. Postoperative surgical outcomes were assessed; quality of life and sexual function were assessed at long term follow-up (>12 months) with 2 scientifically validated questionnaires.

**Methods:**

We conducted a retrospective analysis of a prospectively maintained database. Long-term sexual function and quality of life were assessed by standardized questionnaires. Descriptive statistics were conducted.

**Results:**

8 patients were included in our study, 5 patients responded to quality of life and sexual function analysis. Surgical outcomes were in line with literature: one minor complication (minor dehiscence requiring a skin graft), one major complication (flap loss, requiring a second, contralateral flap) occurred. No reconstructive failure occurred. Average time to complete wound healing was 17 days (SD ±5). Quality of life scores over 70/100 in four out of five categories; social function was rated lowest: patients reported very few residual complaints. Sexual outcome analysis emphasizes the positive impact of the reconstruction. As expected, Fournier's Gangrene heavily affected patient's sexual health.

**Conclusions:**

Perineal reconstruction with ALT shows excellent quality of life, and good sexual health outcomes. Currently the lack of reliable and relatable data leads to the impossibility to compare different reconstructive procedures. We emphasize that assessing sexual function and quality of life after perineal reconstruction is paramount to weigh reconstructive success.

## Introduction

Fournier's Gangrene is a necrotizing soft tissue infection of the perineum, with mortality rates reported as high as 45% (1, 2). Similar to other fulminant necrotizing soft tissue infections, radical debridement with broad spectrum antibiotics is considered the only effective treatment, albeit causing vast soft tissue defects (3). The perineum is a particularly challenging region to reconstruct, given the presence of genito-urinary organs with complex functions to preserve and the proximity of the anus, with therefore increased risk of bacterial contamination. Furthermore, the perineum presents a complex three-dimensional geometry including both convex and concave structures, all involved in dynamic movements.

Reconstructive techniques have been classified over the years following a “reconstructive ladder”, from simplest, e.g. healing by secondary intention or granulation, to most complex, free flap reconstruction. Given the extensive soft tissue defects encountered after Fournier gangrene, specific reconstructive techniques are usually necessary.

Various techniques have been described in perineal reconstruction (4). Skin grafts are invaluable adjuncts to many extensive soft tissue defects, having the advantage of being simple and fast to perform, having and being available to many patients in simple technical settings (5). Grafts do however carry substantial drawbacks in a complex region such as the perineum, both in shape and function. Indeed, the grafted skin usually forms a fibrous, rigid scar with minimal pliability, and remains very sensible to mechanical stress because of its lack of subcutaneous tissue and dermis (6). Moreover, the take rate of grafts in humid, convex/concave areas is oftentimes plagued by graft infection or maceration, leading to partial reconstructive failure (7).

Many disadvantages are avoided using local flaps (8, 9). Albeit more complex, flap surgery offers ample, stable tissue where needed. Literature has reported various donor sites, with a historical preference towards abdominal musculo-cutaneous flaps, namely the vertical rectus abdominis muscle flap (VRAM). Although very potent, it suffers severe abdominal donor site morbidity, with reported herniation rates of up to 66% were described after VRAM harvest (10, 11).

On the other hand, the thigh offers stable, pliable skin with minimal donor site morbidity. The anterolateral thigh flap described by Song has been described in perineal reconstruction from 1992 onwards (12, 13). It allows for a large skin paddle to be harvested and easily reaches the perineum (14). Flaps up to 8 cm allow for primary closure of the thigh and the largest defects can be covered either by larger flaps, or by bilateral flaps if necessary, harvested simultaneously by two teams. If bulk is needed due to particularly aggressive debridement, tailored amounts of vastus lateralis muscle can be harvested to fill dead space along the fascio-cutaneous flap as a combined ALT-VL pedicled flap (14). Harvested muscle has no significant functional impact at long term (15). This ductility made this flap the preferred choice for complex perineal reconstruction in patients who underwent abdomino-perineal resection for oncologic reasons in our institution (16).

The use of ALT flaps after Fournier's gangrene has been described previously; however scarce data describes long-term outcomes, especially functional outcomes (e.g., seating discomfort, walking disorders or chronic pain). Czymek et al. described outcomes and quality of life measures after Fournier's gangrene, however not specifically describing which reconstructive intervention was performed (17). No Fournier-specific study examined perineal reconstruction with thigh flaps measuring quality of life or functional results.

The aim of this retrospective study is to review the functional outcomes and complications in patients undergoing perineal reconstruction after Fournier gangrene with ALT flaps, focusing not only on surgical aspects but also on quality of life, functional and sexual recovery after surgery.

## Patients and methods

### Data gathering and outcome definition

We analyzed the prospectively maintained department database on perineal reconstruction with cases treated 1 January 1999 to 31 January 2021, accounting for 41 reconstructions. Patients who underwent oncological resections were excluded, including in our analysis only patients with histopathologically confirmed signs of necrotizing gangrene of the genitoperineal area (Fournier's gangrene). Out of these, we excluded all patients with superficial wounds treated by split thickness skin graft, patients with excessive skin laxity allowing for fasciocutaneous advancement flaps or local perforator flaps, or patients who were non eligible for ALT flaps (i.e., anterior access hip replacement, femoral fractures, etc …) and therefore reconstructed with other major musculocutaneous flaps such as myocutaneous gracilis or VRAM. Patient selection occurred chronologically, to reduce patient selection bias.

Data was compiled from the institutions' electronic medical records; patient characteristics, comorbidities, defect size, operation duration, postoperative and long-term complications as well as follow-up duration were assessed.

Surgical outcomes were defined as described in previous series characterizing perineal reconstructions by ALT-VL flap (16). We recorded flap related complications, defined as “major” in case of total or partial flap loss, major wound dehiscence at donor or recipient site involving more than a third of the incision length and persistent perineal dead space requiring additional reconstructive surgical procedures during follow-up. Minor complications comprised local infections resolving by antibiotic therapy alone, seroma or hematoma not requiring drainage, dehiscence at donor or recipient site involving less than a third of the incision length that healed with conservative treatment, debridement, split-thickness skin graft (STSG) or flap advancement. We defined “wound healing” as intact scar/skin, and considered the total healing time if the patient underwent a synchronous secondary surgical procedure.

### Functional outcomes

We analyzed long-term outcomes (i.e., >12 months after the initial procedure). Exclusion criteria were defined as loss of follow-up, refusal or inability to answer the questionnaires. We selected two functional questionnaires drafted by the EORTC (European Organization for Research and Treatment of Cancer): QLQ C30 V3 and QLQ-SHQ C22 V1 for global quality of life and sexual health, respectively. QLQ C30 is well established in oncological quality of life studies (18), and SHQ is in the final validation stages for sexual quality of life assessment (19). Although validated in oncological patients undergoing colorectal operations, the similar issues faced by the patients we reconstructed (i.e., large perineal defects, complex recovery, potential loss of function) are well examined by both these questionnaires. Scoring and data analysis was performed according to the published questionnaire summary (19, 20).

Institutional written consent was obtained from all patients regarding retrospective use of data for scientific studies, including use of photographic documentation. The study was designed according to the guidelines of the 1975 Helsinki Declarations.

### Surgical technique

Serial debridement was performed until stabilization of the patient in order to obtain a clean, stable wound. Debridement focuses on suprafascial structures, i.e., skin and subcuticular fat; however, cases with progressive infection involving the abdomen needed more aggressive debridement. After obtaining a clean wound, the size and localization of the defect lead to patient selection for flap reconstruction.

Preoperative flap planning was conducted as previously described (14). Markings identifying ALT-perforators by pencil Doppler are performed prior to the reconstruction; patients are placed in lithotomy position in order to access both the thigh and the perineum. Dissection of the ALT perforator flap was converted, when needed to composite ALT-VL flap as described in previous publications (14, 16). This technique allows for a tailored harvest of both coverage, (skin), bulk, (muscle) and eventually tensile component such as fascia lata, allowing reconstruction of the penile suspensory ligament (21).

Once elevated, the flap passes through a subcutaneous tunnel under the sartorius and rectus femoris muscles until reaching the perineum: this leads to a gain of 5 to 8 centimeters and avoids tension on the pedicle (16). Flap inset was performed in layers with deep sutures of the fascia using Vyril 2-0, inverted subcuticular sutures using Vycril 2-0 and Prolene 3-0 suture to the skin.

Postoperative monitoring was performed both clinically and using pencil Doppler. Patients were at bed rest alternating lateral and supine position. Mobilization was started by postoperative day 5, while seating was allowed after full healing and suture removal, usually 3 weeks postoperatively.

### Statistical analysis

Descriptive statistics of the patient population, defects to reconstruct and flap characteristics were performed. Data were expressed as mean (± standard deviation). Data compilation and descriptive statistics were conducted on Excel (Microsoft Corp., Redmond, WA, USA).

## Results

After applications of exclusion criteria, 8 patients who received a reconstruction after Fournier Gangrene using 9 pedicled ALT flaps during the study duration were included in our study. Table 1 describes patient characteristics.

**Table 1:** Patient characteristics.

Patients characteristics	
Patients	8
Flaps	9
Mean age (SD)	50 (±14.5)
Comorbidities	
Smoking	8
Chronic alcohol	5
Diabetes Type II	2
Mean BMI (kg/m^2^)	24.9 (±3.4)
Follow-up, months (SD)	19 (6.2)

Note the young average age of the patients. In this series, all patients were male. Surgery-relevant comorbidities are stated, with all patients in the series smoking, and a high incidence of chronic alcohol abuse.

Concerning quality of life evaluation and sexual health, we could asses 5 patients out of 8 in the group under study: two patients deceased at time of evaluation and one patient unable to reply because of cognitive impairment. One patient was lost to long term follow-up. Response rate in the remaining 4 questionnaires was 100%, no items were excluded.

### Surgical outcome analysis

Patient's characteristics are stated in Table 1. Interestingly, all patients were smokers. Alcohol abuse was prevalent in 5 patients, and diabetes type II in 2 patients. Mean BMI was 24.9 kg/m^2^ (± 3.4). Mean follow-up was 19 months. Outcomes were measured both during the initial hospitalization and during follow up. Defect size to reconstruct, isolated pathogen, patient comorbidities and complications, as well as the time required for complete healing are listed in Table 2. We recorded one minor complication (patient n.4), requiring debridement and split thickness skin grafting after minor dehiscence.

**Table 2:** Patient data.

Patient	Flap	Defect area (cm^2^)	Germ	Complication	Co-Morbidities	Time to heal
1	1	128	Strep. milleri, Bact. fragilis	none	Smoking, chronic alcohol abuse, diabetes	14
2	2	130	Serratia marescens, Enterobacter cloacae	none	Smoking	12
3	3	140	Strep. pyogenes group A	none	Smoking, chronic alcohol abuse	14
4	4	140	Ent. faecalis	minor dehiscence, skin graft	Smoking, chronic alcohol abuse	21
5	5	160	Ent. faecalis, Enterobacter cloacae, E. coli	flap loss	Smoking	n/a
5	6	160	Ent. faecalis, Enterobacter cloacae, E. coli	none	Smoking	17
6	7	192	E. coli, Beta hemol. strep., Fusobacterium	none	Smoking, chronic alcohol abuse, diabetes, obesity	25
7	8	240	E. coli, K. pneumoniae	none	Smoking, chronic kidney failure	21
8	9	120	E. Coli, S. pyogenes	none	Somking, Hepatitis C, Cirricis Child B	14

We performed nine flaps in eight patients, with one flap failure (patient five) requiring a second flap of the contralateral thigh. We did not note any reconstructive failure. One patient suffered a minor complication with minor dehiscence (patient four), treated by split thickness skin graft. Note the high incidence of smoking in this series (all patients).

One patient (patient n.5) suffered a major complication after total flap loss due to venous congestion of the flap leading to pedicle thrombosis. Prompt revision in the operating theatre did unfortunately not resolve the issue, and the flap was lost. The voluminous defect (160 cm^2^) required reconstruction with a second, contralateral flap with uneventful healing.

**Figure 1:**
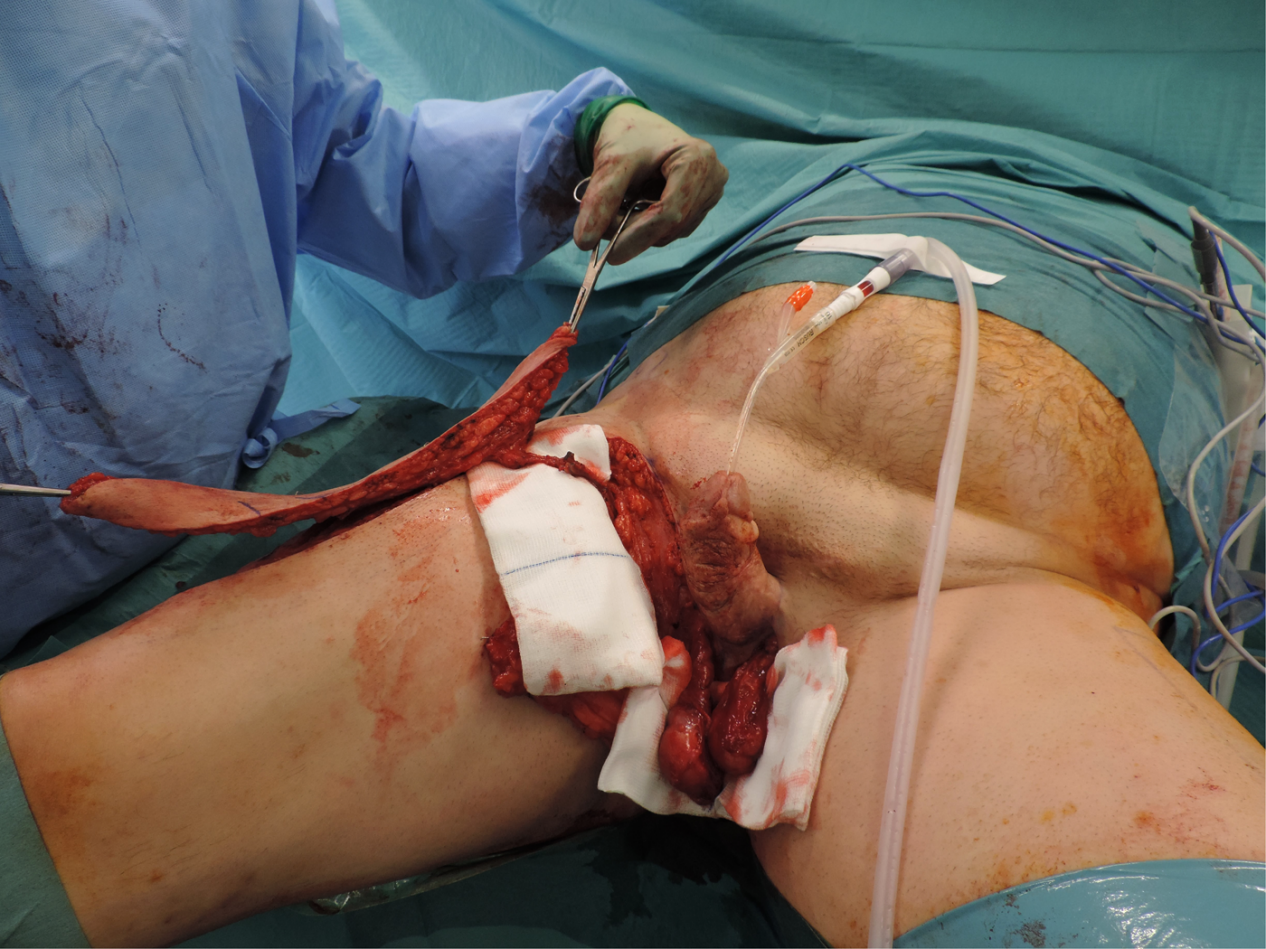
Typical outcome of perineal reconstruction after Fournier’s gangrene. In this case, the pedicled anterolateral thigh flap was used to reconstruct both the perineum and scrotum. The flap is tunneled under the Sartorius in order to reach the perineum.

**Figure 2:**
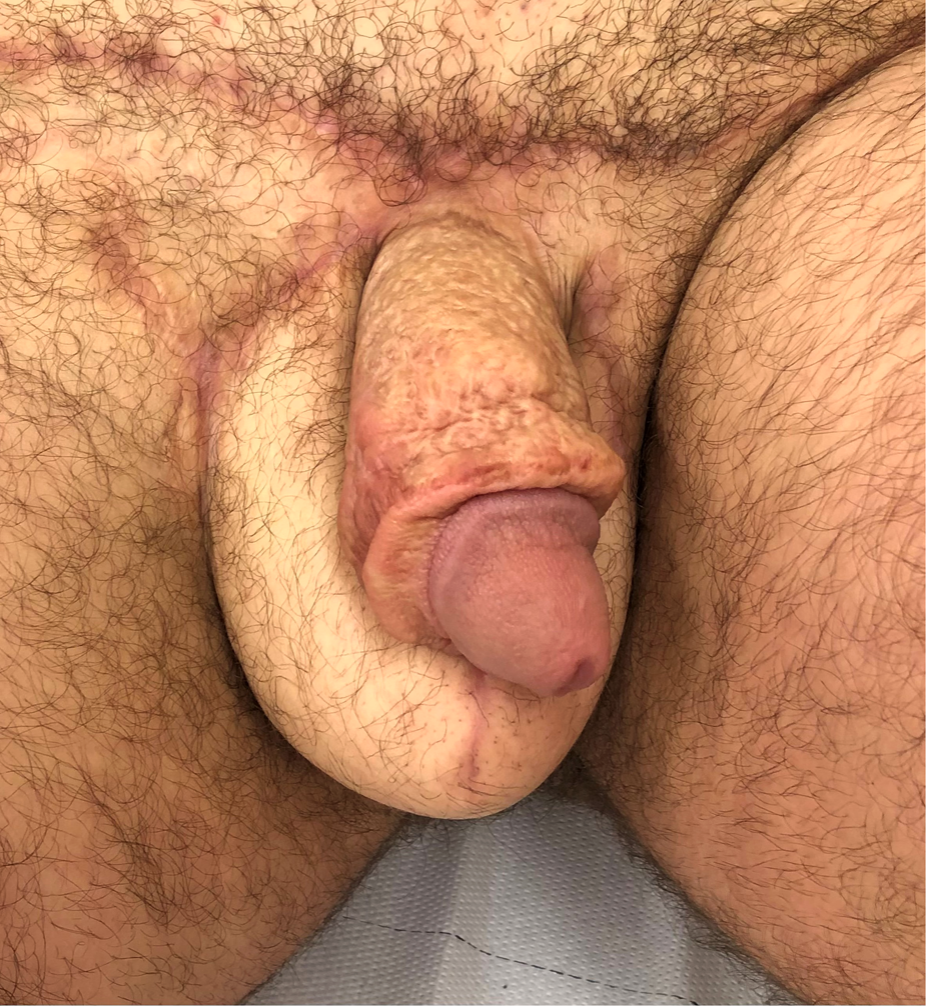
9 Months postoperative view of the same patient. Note excellent mimicry of the thigh skin to a native scrotum.

Time to complete wound healing was in average 17 days (SD ±5) prior to total stitch removal. We did not record any long-term complications in this series: patients were specifically asked for seating discomfort, sciatic or sacral decubitus ulcers, walking disorders or chronic pain both of the perineum and at the donor site. Figure 1 depicts the intraoperative view of perineal and scrotal reconstruction case. Figure 2 shows the same patient at 9 months follow-up.

### Functional outcome analysis

Quality of life scores show excellent functionally (i.e., confidence in erection, satisfaction of sexual life), with mean scores over 70/100 in four out of five categories; social function was rated lowest, although still above average with a mean of 62/100 (Table 3). Patients reported very few residual complaints in the symptom scales. Interestingly, many patients reported financial difficulties even long after the initial surgical procedure.

**Table 3:** Quality of life and sexual health questionnaires.

Functional scales	Mean	Range	*N*
**Quality of Life – QLQ C30**			
Global health status	77	67–83	4
Physical functioning	75	53–93	4
Role functioning	75	50–100	4
Emotional functioning	71	42–92	4
Cognitive functioning	92	67–100	4
Social functioning	62	50–100	4
Symptom scales			
Fatigue	17	0–33	4
Nausea and vomiting	4	0–17	4
Pain	8	0–33	4
Dyspnea	8	0–33	4
Insomnia	17	0–33	4
Appetite loss	8	0–33	4
Constipation	33	0–67	4
Diarrhea	8	0–33	4
Financial difficulties	33	33	4
**Sexual Health Questionnaire SHQ C22**			
Sexual satisfaction	46	29–70	4
Importance of sexual satisfaction	67	33–100	4
Libido	33	0–67	4
Impact of treatment on sexual life	8	0–33	4
Communication with professionals	16	0–33	4
Insecurity with partner	42	33–67	4
Confidence of erection	58	0–100	4
Masculinity	42	0–67	4
Symptom scales			
Sexual pain	44	22–78	4
Worrying about incontinence	33	0–100	4
Fatigue	33	33–67	4

Functional scores given in points out of 100 (high mark), higher is better. Symptom scores given as occurrence in percent (high occurrence), lower is better. n: number of patients.

Sexual outcome analysis emphasizes the positive impact of the reconstruction, with patients reporting a moderately high score (67/100) in their sexual satisfaction. Interestingly, libido scores were relatively low (33/100), especially in comparison to higher scores in confidence of erection (58/100) and importance of sexual satisfaction (67/100). Importantly, patients reported a low score in communication of their sexual health with their tending physicians (16/100), underlining the need to address this issue in specialist consultation.

As expected, Fournier's Gangrene heavily affected patient's sexual health, with a low score on this item (8/100). Residual symptoms (pain, incontinence, fatigue) seemed to occur moderately seldom (33–44/100).

## Discussion

Analysis of this series showed extremely satisfactory outcomes, both surgical and in terms of quality of life, underling the essential role of plastic surgery reconstruction after Fournier's gangrene. Although complication rates seem high in comparison with other reconstructive procedures, they align to literature describing perineal reconstruction (11, 14, 16). Moreover, we could note a possible skew due to a complete flap loss in the early stages of the learning curve of our team.

Unlike skin grafts, the ALT offers stable skin allowing much better pliability required to reconstruct a complex three-dimensional anatomical region. Furthermore, its versatility in its ability to include several different tissue types (i.e., muscle, fascia lata) can be a clear advantage in complex reconstructions, such as scrotal reconstruction, penile suspensory ligamentoplasty or filling deep perineal dead space (16, 21).

Its donor site is usually unproblematic, as opposed to abdominal flaps where herniation and bulging are commonplace (11), seldom requiring a skin graft in the largest flaps (over 8–10 cm in width, depending on the patient physiognomy).

### Surgical outcomes

In this series, we do note that the complexity of the procedure imposes a learning curve, with an early case suffering complete flap loss. The latter however remain rare, literature stating a flap loss rate under 4% (22). Moreover, the small sample size is more susceptible to single major complications skewing the global outcomes. Complication rates are in line with current literature, and large series of pedicled ALT flaps performed in our institution report similar surgical outcomes (14, 16).

The main advantage of the ALT is the coverage achieved with one operation, showing a particular ductility due to pedicle length and possibility to harvest as purely perforator, or combined fascia or muscle component when necessary. This is a clear advantage and offers as it can be tailored to precisely restore form and function, from creation of a scrotal sac to reconstruction of a suspensory ligament. Where grafts in the perineum usually require extensive physiotherapy and ergotherapy to achieve stable, supple coverage, the ALT offers immediate pliability and better function, minimizing scarring, retraction and chronic wounds.

Even if the ALT has become a workhorse for complex reconstructions, and in our institution it has imposed itself as a reliable and versatile, surgical outcomes alone do not depict the full picture. Functional assessment of patient-related outcomes after reconstruction of perineum and/or genitalia remains a critical point when judging of the effectiveness of reconstruction.

### Patient related outcomes

Modern reconstructive surgery restores function and form, and a simple visual analysis of long-term results lacks to assess the actual function of the surgeons work. In this small series, we note the excellent outcome in functional quality of life. Although some items (i.e., moderately lower social function) do score lower, the global quality of life is good despite a life threatening condition in oftentimes polymorbid patients and complex subsequent reconstruction. We infer that the sexual wellbeing can play a major role in both emotional and social health, and may cause lower scores in the functional scales of quality of life. Very few long term symptoms were reported: occasional constipation was one of them but did not seem to relate to the surgical procedure (debridement/reconstruction); notwithstanding constipation being a frequent symptom in the general population, with an incidence of up to 15% worldwide (23). Financial difficulties occurred occasionally, generally matching with the patient's social state.

Sexual health analysis showed a lower global score when compared to global functional parameters, however patients reported residual sexual activity, with moderately high sexual satisfaction (46/100), and above average confidence in their erection (58/100). Fournier's gangrene understandably affected their sexual life negatively (scoring 8/100), however the lack of control group with other means of reconstruction does not allow comparison with other means of reconstruction such as skin grafts, or other flap techniques.

Interestingly, a discrepancy between reported fatigue in QoL and SHQ (17 vs. 33/100) may denote a higher impact of fatigue on sexual health than general quality of life. This could explain globally lower scores in SHQ vs. QoL, since sexual function depends heavily on a good perineal form and function. We infer that the SHQ is an excellent measure of reconstructive success in perineal reconstruction.

Limitations of this study include a small series, with possible confounding bias due to the lack of good quality of life and sexual health data in a comparable, healthy group. This emphasizes the need to systematically assess outcomes in both the general population and after reconstructive surgery. Moreover, our patient population being exclusively male, depicting the increased incidence in male, results do not reflect neither quality of life nor sexual health in women. We do concede the difficulty to extrapolate trends in the small population under study, however the available data is meaningful in assessing function of the patient in his everyday life.

## Conclusions

To our knowledge, this is the only study that assesses functional Quality of Life and Sexual Health outcomes in perineal reconstruction after Fournier's gangrene. The ALT shows excellent quality of life outcomes, and moderately good sexual health. This is however the only data that is available after reconstructive surgery after a major sort tissue infection, the lack of reliable, relatable data leads to the impossibility to compare different reconstructive procedures. Our results show that assessing sexual function after reconstructive surgery of the perineum is paramount to analyze both function and form.

Furthermore, the ALT flap offers ample stable, pliable skin to reconstruct the perineum, with good results and complication rates in line with current literature. It is a valid alternative to abdominal flaps without potential donor site comorbidities.

## Data Availability

The raw data supporting the conclusions of this article will be made available by the authors, without undue reservation.
