# Erratum: Quality of life and sexual health after perineal reconstruction in Fournier gangrene using pedicled anterolateral thigh flaps

**DOI:** 10.3389/fsurg.2023.1176610

**Published:** 2023-03-10

**Authors:** Frontiers Production Office

**Affiliations:** Frontiers Media SA, Lausanne, Switzerland

**Keywords:** Fournier Gangrene, reconstruction, pedicled anterolateral thigh flap, perineal defect, sexual wellbeing, quality of life

An Erratum on Quality of life and sexual health after perineal reconstruction in Fournier gangrene using pedicled anterolateral thigh flaps By Rossi SA, de Schoulepnikoff C, Guillier D, Raffoul W and di Summa PG. (2022) Front. Surg. 9:994936. doi: 10.3389/fsurg.2022.994936

An omission to the funding section of the original article was made in error. The following sentence has been added: “Open access funding was provided by the University of Lausanne”.

The original version of this article has been updated.

